# Efficacy of different revision procedures for infected megaprostheses in musculoskeletal tumour surgery of the lower limb

**DOI:** 10.1371/journal.pone.0200304

**Published:** 2018-07-05

**Authors:** Irene Katharina Sigmund, Jutta Gamper, Christine Weber, Johannes Holinka, Joannis Panotopoulos, Philipp Theodor Funovics, Reinhard Windhager

**Affiliations:** 1 Department of Orthopaedics and Trauma Surgery, Medical University of Vienna, Vienna, Austria; 2 Section for Medical Statistics, Medical University of Vienna, Vienna, Austria; Van Andel Institute, UNITED STATES

## Abstract

**Purpose:**

The incidence of recurrent infections in patients following one or two stage revision for infected megaprostheses after resection of bone tumours was investigated. The difference between retaining at least one well fixed stem and a complete removal of the megaprosthesis during a two stage revision was also analysed.

**Methods:**

627 patients who experienced a replacement of a musculoskeletal tumour by megaprostheses were recorded. An infection occurred in 83 of 621 patients available for follow-up. 61 patients underwent one stage revision, and 16 patients two stage revision for the first revision surgery. In the entire study period, two stage revision was performed 32 times (first, second, and third revision).

**Results:**

The cumulative incidence analysis showed a reinfection probability after one stage revision of 18% at one year, 30% at two years, 39% at five years, 46% at ten years, and 56% at 15 years. After two stage revision, a reinfection probability of 28% at two years, and 48% at five years was calculated. Cumulative incidence curves did not differ significantly (Gray’s test; p = 0.51) between one and two stage revision (with and without complete removal of the stems). In two stage revision (n = 32), a statistically significant difference in infection rates between patients treated with complete removal of the megaprosthesis (n = 18) including anchorage stems and patients with at least one retained stem (n = 14) was shown (Fisher’s exact test, p = 0.029).

**Conclusion:**

Two stage revisions with complete removal of the megaprosthesis showed the best results among limb salvage procedures for the treatment of infected megaprosthesis.

## Introduction

Due to improved prognosis of patients with bone tumours, limb salvage has become of primary concern in orthopaedic tumour surgery. In the literature, the overall survival rate of megaprostheses was demonstrated to be 79 to 87% at five years, 71 to 80% at 10 years, and 56% at 15 years [1, 2]. However, the complication rate is high compared to total joint replacement after osteoarthritis. While the prosthetic joint infection rate after a routine total joint replacement was reported to be 1 to 2% [3], the infection rate after primary limb salvage was calculated to be eight to ten times higher at 8 to 15% [4–9]. This problem is likely to prolong operating time, extensive soft tissue dissection, immunosuppression, and adjuvant treatment [10]. Furthermore, Capanna et al. [11] described a reinfection rate of 43%. Hence, an appropriate approach for the correct treatment of septic megaprosthesis is of immense importance to achieve infection-free conditions. Surgical treatment options for periprosthetic joint infections (PJI) after conventional total joint arthroplasty (TJA) are debridement, resection arthroplasty, arthrodesis, one stage revision (with complete exchange of the prosthesis), two stage revision (with complete removal of the prosthesis), and amputation. While debridement showed very low rates of eradication in oncological patients [12], arthrodesis or resection arthroplasty are not feasible in patients with large bone defects. Hence, one stage revision and two stage revision are appropriate procedures for limb salvage in oncological patients to control deep infections. However, due to well osseointegrated stems, a complete removal of the uncemented megaprosthesis is occasionally linked with vigorous attempts at extraction and increased bone loss during the operation. In the first decades after introduction of megaprostheses, a complete removal was therefore not always performed during a one stage or two stage revision. Nevertheless, despite the diversity of treatment modalities, reinfections, which are even more difficult to treat, still arise [11, 12].

The aim of this study was to assess the incidence of reinfection and re-reinfection in patients following one stage or two stage revision for infected megaprostheses after resection of bone tumours of the lower limb. We compared both surgical strategies with each other. Furthermore, we investigated the difference between retaining at least one well ingrown stem and a complete removal of the megaprosthesis during a two stage revision.

## Material & methods

### Study design

This retrospective cohort study was conducted in a tertiary healthcare centre. Data were collected from the prospectively enrolled Bone and Soft Tissue Tumour Registry. The study was approved by the institutional review board (Ethical Review Board—Medical University of Vienna—EK1817/2016) and done in accordance with Declaration of Helsinki. All patient information and records were anonymized and de-identified prior to analysis.

### Study population

From 1982 until 2017, 627 patients with a primary replacement of a musculoskeletal tumour of the lower limb and reconstruction by a megaprosthesis were recorded. Six patients (1%) were excluded because of inadequate follow up. Megaprostheses, which were used to reconstruct large segmental defects, included the KMFTR (Kotz Modular Femur and Tibia Reconstruction System, Howmedica GmbH, Kiel, Germany), the HMRS (Howmedica Modular Reconstruction System, Howmedica GmbH, Kiel, Germany), the GMRS (Global Modular Reconstruction System, Stryker Corp., Mahwah, NJ), and the MUTARS (Modular Universal Tumour and Revision System, Implantcast, Buxtehude, Germany).

### Definition of infection

**Fig 1:**
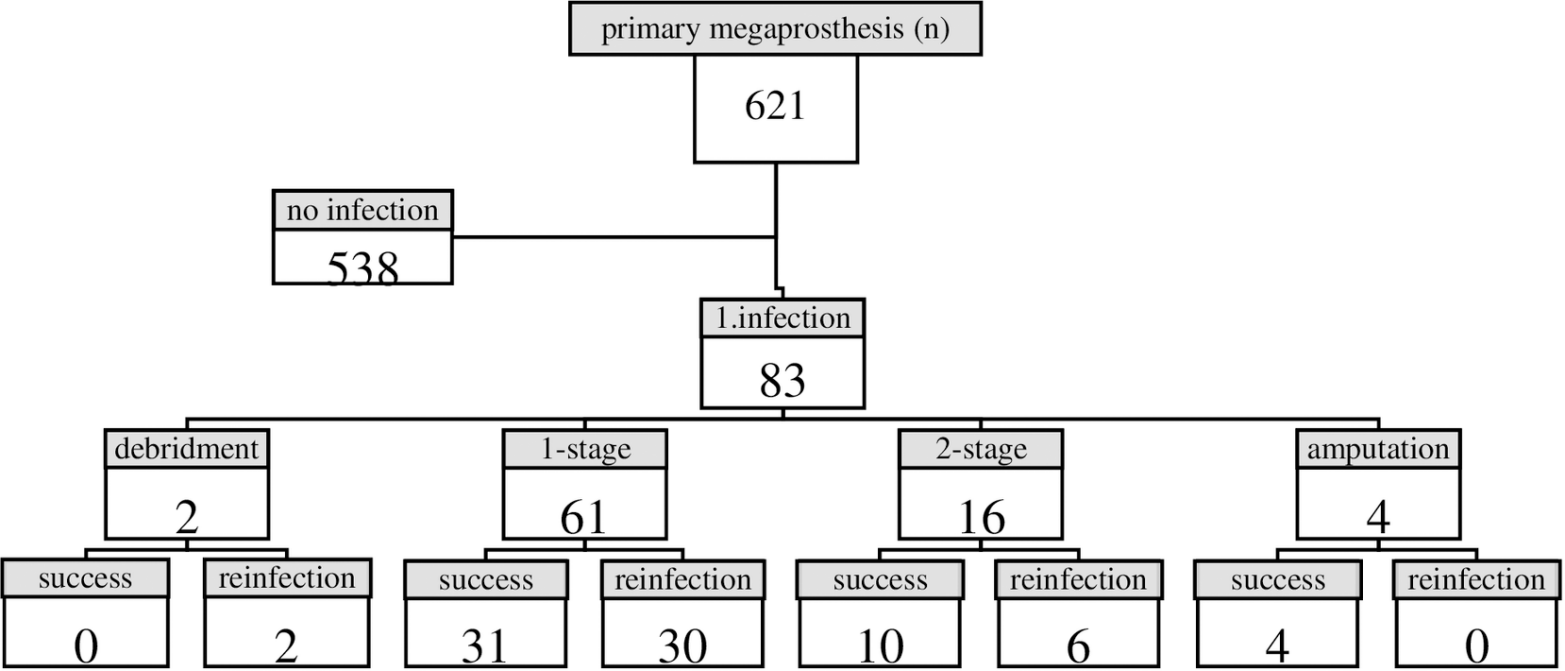
Flow chart of all patients with infection following a primary replacement of a musculoskeletal tumour of the lower limb and reconstruction by a megaprosthesis.

In this study, we were unable to use new infection classification systems such as the Musculoskeletal Infection Society (MSIS) criteria because newer diagnostic methods (e.g. white blood cell count in the synovial fluid, percentage of polymorphonuclear neutrophils, leukocyte esterase test strips) were not performed in the first twenty years of the study period. Therefore, a megaprosthesis was deemed septic when either one or more of the following criteria were present: (i) a fistula, (ii) a positive microbiological culture, (iii) periprosthetic pus, and/or (iv) definitive histological evidence of an infection. According to the systems of Coventry [13] and Fitzgerald [14], the cases were categorized as follows: Class-I, in which an infection was present within one month after primary surgery, Class-II, between one month and 24 months, and Class-III, more than 24 months after the operation. In our study cohort, 83 cases (infection rate: 13.4% [95% CI: 10.8–16.4%]) were classified as megaprosthesis joint infections (MJI). Fig 1 shows a flow chart representing the entire study population.

### Surgical procedures

In two patients (2.4%), a debridement was performed because the infection occurred within the first month after primary implantation. An amputation was performed in four patients (4.8%) with extensive osteolysis, loosening of the prosthesis, and poor soft tissue coverage.

In the first decades after introduction of megaprostheses, one stage revision surgery was the preferred primary procedure for infection control in our institution. Over the last years, two stage procedures have been advocated [12, 15–18] as the method of choice for patients with PJI. Hence, they have replaced one stage revision surgery at our institution. Therefore, there should be no selection bias in favour or against one stage or two stage revision.

Sixty-one patients (73%) underwent a one stage revision. All exchangeable components and polyethylene parts with exception of the anchorage stems were removed. A thorough debridement and rinsing with povidone iodine (Betaisodona®; Mundipharm, Limburg/Lahn, Germany) was performed. A pulse lavage was carried out when available. The wound was packed with povidone iodine sponges and provisionally closed before repeated sterile washing and covering. The instruments, the gowns and glows of the operating team were exchanged. Thereafter, the sponges were removed and another rinsing of the wound and cleaning of the anchorage components were completed. Finally, the new components were implanted.

Sixteen patients (19%) underwent a two stage revision. In seven patients, the megaprosthesis was completely removed. In nine patients, at least one well ingrown stem remained in situ due to stability despite vigorous attempts at extraction. A thorough debridement, rinsing with povidone iodine and pulse lavage was done and a temporary antibiotic-loaded bone cement spacer inserted. After antimicrobial treatment and a mean time of 116 days (range: 19–401 days), the second procedure was performed. The spacer was explanted and another thorough debridement, rinsing with povidone iodine and pulse lavage was completed. A new megaprosthesis or new components were implanted.

The demographic data of this cohort is shown in Table 1. The overall median follow-up was 125 months (range: 13–423 months). Four patients died within the first two years (range: 13–17 months). In all other patients, the follow-up was at least 2-years.

**Table 1:** Demographic data of 83 patients with infected megaprostheses.

Demographic parameter	n (%)
Age (range)	32.3 (9–86)
Gender	
Female	39 (47)
Male	44 (53)
Tumour entity	
Osteosarcoma	51 (62)
Ewingsarcoma	7 (8)
Chondrosarcoma	7 (8)
Soft tissue sarcoma	13 (16)
Bone metastases	5 (6)
Site of reconstruction	
Proximal femur	12 (15)
Distal femur	42 (51)
Proximal tibia	26 (31)
Total femur	1 (1)
Total knee	2 (2)
Type of megaprostheses	
KMFTR	55 (66)
HMRS	11 (13)
GMRS	16 (19)
Repiphysis	1 (1)
Adjuvant treatments	
Local radiotherapy	18 (29)
Chemotherapy	63 (76)
OP-procedure	
Debridement	2 (2)
1-stage	61 (74)
2-stage	16 (19)
Amputation	4 (5)

KMFTR = Kotz Modular Femur and Tibia Reconstruction System, HMRS = Howmedica Modular Reconstruction System, GMRS = Global Modular Reconstruction System.

### Statistical analysis

Data are summarized as mean and range for continuous variables, and absolute and relative frequencies for categorical variables. Reinfection rates were calculated for one stage and two stage revisions. The difference between both surgical methods were assessed by a chi-squared test.

A competing risks survival analysis was performed to analyze the time to reinfection after the first revision and time to re-reinfection after the second revision with death and reinfection as competing risks. Cumulative incidence rates for both events were calculated as the probability of experiencing one event before a given time and before experiencing the other event. Cumulative incidence curves were drawn for one and two stage revision procedures.

By calculating a univariate cause-specific proportional hazard ratio, we assessed the influence of different risk factors on reinfection. Hazard ratios were calculated for each model. For metric covariates, the hazard ratio indicates the multiplicative change of the hazard per one unit. In the case of categorical covariates, the hazard ratio shows the hazard ratio of each group in comparison to the reference group. The significance level for all tests was 5%. Analysis was done using the open source software R 3.3.2 and the packages “survival” and “logistf”.

## Results

### Reinfection

Overall reinfection rate after one stage revision and two stage revision was 49% and 38%, respectively. A detailed list is shown in Table 2. There was no statistically significant difference in reinfection rates between the different surgical procedures (Chi-squared test, p = 0.43).

**Table 2:** Reinfection rate of the different op procedure: The total number of patients (N), the number of reinfections (n), the reinfection rate (%), and the lower and upper bounds of the 95% confidence interval (95% CI) for the reinfection rate separately for each op procedure. 2-stage revision = patients with and without complete removal of the well fixed stems for the first revision surgery.

OP procedure	N	Reinfection (n)	Reinfection Rate (%)	95% CI
Debridement	2	2	100%	20–100%
1-stage	61	30	49%	36–62%
2-Stage	16	6	38%	6–76%
amputation	4	0	0%	-

Both patients treated with debridement experienced reinfection after 2.3 and 43.5 months, respectively. The first patient underwent a one stage procedure after reinfection, and the second patient a two stage procedure. Afterwards, both patients were free of infection for a period of 62 months each. During follow up, one patient died from metastatic renal cell carcinoma.

All four cases treated with an amputation were infection free after the surgical procedure (0% reinfection rate); none of the four died during follow up.

**Fig 2:**
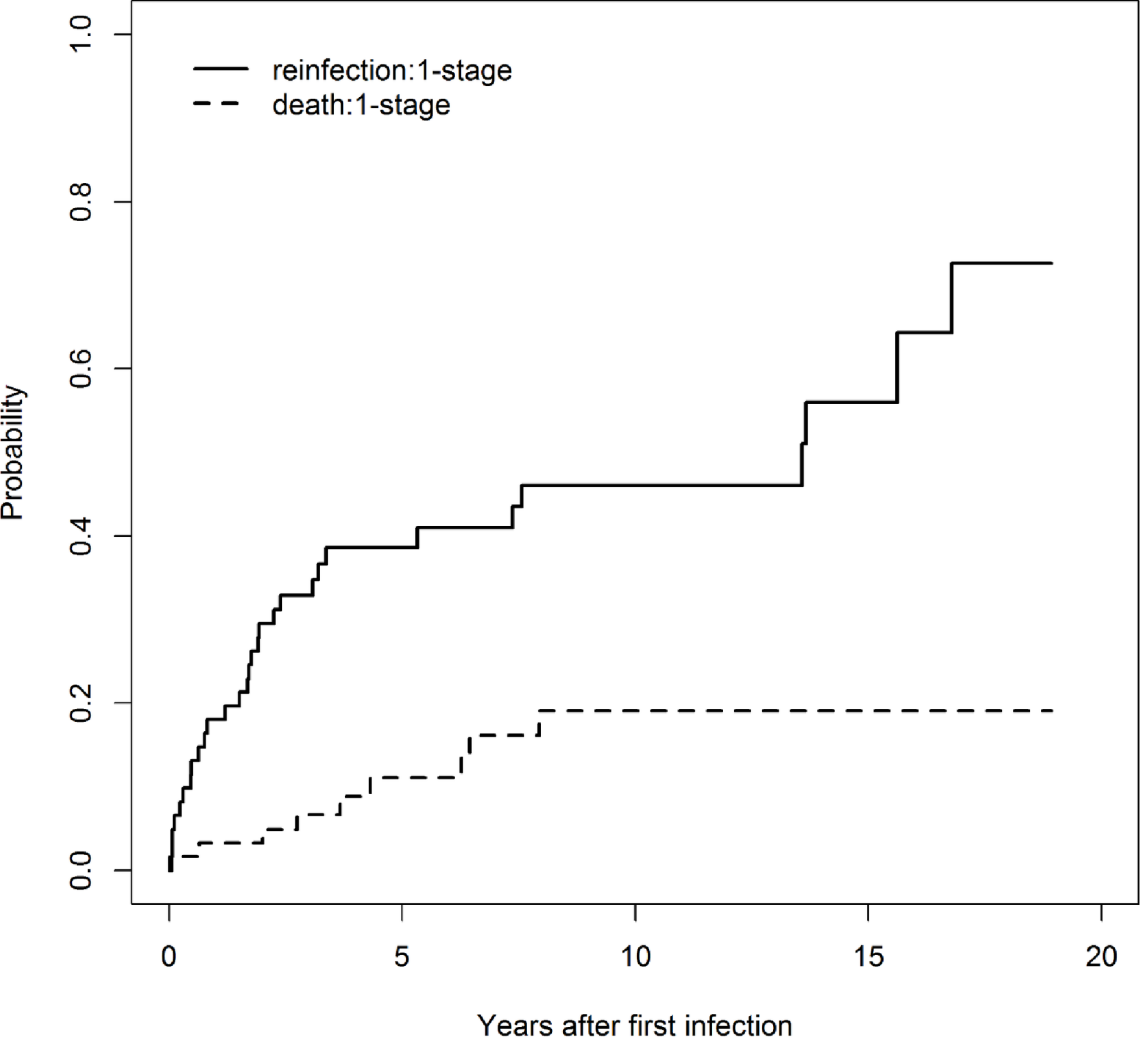
The cumulative incidence of reinfection (continuous line) and death (dashed line) after one-stage revision.

The cumulative incidence analysis showed a reinfection probability after a one stage procedure of 18% (CI 95%: 8–27%) at one year, 30% (CI 95%: 17–40%) at two years, 39% (CI 95%: 25–50%) at five years, 46% (CI 95%: 31–58%) at ten years, and 56% (CI 95%: 36–70%) at 15 years. In Fig 2, the cumulative incidences curves are shown. Reinfection occurred after a mean of 43 months (range: 0.7–201 months). Four patients died from metastatic disease and four for reasons unrelated to infection after one stage revision without further sign of infection. One patient died from sepsis due to pseudomembranous colitis.

**Fig 3:**
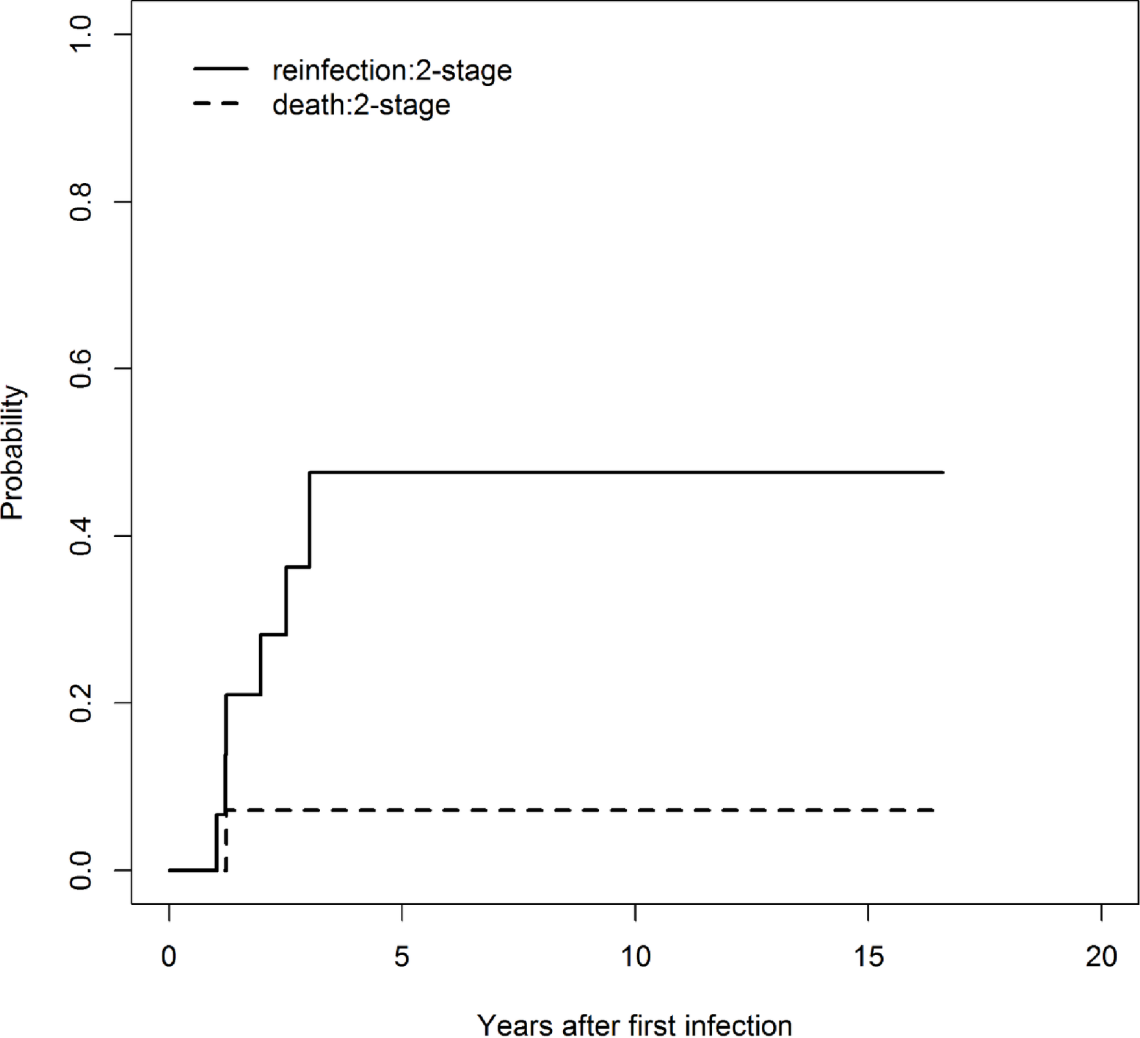
The cumulative incidence of reinfection (continuous line) and death (dashed line) after two-stage revision (n = 16; patients with and without complete removal of well-fixed stems).

In the two stage revision cohort (Fig 3), no reinfection occurred in the first year (0%). After two years, the probability of reinfection rate was 28% (0.4–48%). At five years, the probability of reinfection was estimated at 48% (CI 95%: 9–70%). After approximately three years, no further reinfection occurred. Hence, the estimated probability of reinfection remains constant at 48% (CI 95%: 9–70%) at 10 and 15 years. Reinfection occurred after a mean of 16 months (range: 6–28 months). One patient died from metastatic disease.

Cumulative incidence curves did not differ significantly between one and two stage revision (Gray’s test; p = 0.51). The reinfection rate depending on the localisations of the megaprostheses and surgical procedures is shown in Table 3. No statistically significant difference between localisation and one and two stage revision was detected (Fisher's exact test; p = 0.2258). Furthermore, no statistically significant differences between reinfection rate and localisation after one stage revision (Fisher's exact test; p = 1) and after two stage revision (Fisher's exact test; p = 0.169) were found.

**Table 3:** The reinfection rate (RR) depending on the localisations of the megaprostheses and OP procedures.

localisation	N°	Debridement	RR (%)	1-stage	RR (%)	2-stage	RR (%)	amputation	RR (%)	R	total RR (%)
proximal femur	12	1/1	100	4/8	50	1/1	100	0/2	0	6	50
distal femur	42	1/1	100	17/35	49	1/6	16.7	0		19	43
proximal tibia	26	-		8/16	50	3/8	37.5	0/2	0	11	42
total femur	1	-		0/1	0	-	-	-		0	0
total knee	2	-		1/1	100	1/1	100	-		2	100
total	83	2/2	100	30/61	49	6/16	38	0/4	0	38	46

At the time of second revision, the most common isolated microorganisms were coagulase negative Staphylococci (n = 15) followed by *Staphylococcus aureus* (n = 6 [MRSA; n = 1]), *Klebsiella pneumoniae* (n = 2), *Propionibacterium acnes* (n = 1), *Enterobacter cloacae* (n = 1), *Enterococcus faecalis* (n = 1), *Enterococcus faecium* (n = 1), and *Escherichia coli* (n = 1). This is in concordance with other published series [12, 19–21].

Table 4 shows the cause specific hazard ratios (HR) for the cause reinfection from patients (n = 77) after one stage and two stage revisions in accordance with different factors. The global effects of tumour entity (p = 0.805), location of tumour (p = 0.321), and type of prosthesis (p = 0.36) were tested by a likelihood ratio test and were not significant. The global effect of the infection classification was found to be significant (p = 0.0317). In summary, none of the covariates except the infection classification had a significant effect on reinfection. There is a significantly lower risk of reinfection for Class II compared to Class I (HR = 0.1972, 95% CI: [0.059–0.66], p = 0.0082).

**Table 4:** Cause-specific hazard ratios (HR) and 95% confidence intervals (95% CI) for reinfection from patients after 1-stage and 2-stage (with and without well fixed stems for the first revision surgery) revisions in accordance with different factors. OSA = osteosarcoma, dist = distal, prox = proximal, KMFTR = Kotz Modular Femur and Tibia Reconstruction System, HMRS = Howmedica Modular Reconstruction System, GMRS = Global Modular Reconstruction System, CHT = chemotherapy, RTX = radiotherapy, Infection classification by Coventry and Fitzgerald [13, 14].

Variable	HR	95% CI	p-value
Age (at surgery)	0.98	0.96–1.00	0.07
Female vs Male	0.82	0.42–1.59	0.55
Tumour entity			
Ewingsarcoma vs OSA	0.72	0.21–2.43	0.59
Chondrosarcoma vs OSA	0.75	0.25–2.24	0.61
Soft tissue Sarcoma vs OSA	0.55	0.19–1.59	0.27
Bone metastases vs OSA	0.97	0.13–7.38	0.98
Site of reconstruction			
dist femur vs prox femur	0.69	0.25–1.92	0.48
prox tibia vs prox femur	1.15	0.40–3.29	0.79
femurdiaphysis vs prox femur	4.76	0.52–43.24	0.17
Megaprostheses			
HMRS vs KMFTR	0.51	0.18–1.47	0.21
GMRS vs KMFTR	0.52	0.16–1.72	0.28
CHT vs no CHT	1.32	0.61–2.83	0.48
RTX vs no RTX	1.08	0.49–2.38	0.85
Infection classification			
Class II vs Class I	0.19	0.06–0.66	0.01
Class III vs Class I	0.38	0.12–1.16	0.09
OP procedure			
2-stage vs 1-stage	0.95	0.39–2.30	0.91

Nine (11%; [all one stage revisions]) of the 83 patients were treated with a Ligament Advanced Reinforcement System (LARS®) reconstruction during their first revision surgery. Among these, four (44%) had a reinfection. No statistically significant difference was calculated between patients with and without a LARS® reconstruction (Fisher’s exact test; p = 1.000). In the second and third revision surgeries, no LARS® band or tube was used.

### Re-reinfection

Overall, 38 patients (45.8%) showed reinfection. Of these, three (8%) were treated conservatively with antibiotics to achieve infection suppression: In one patient, no further operation was performed due to reduced general condition. Two patients rejected a second revision due to infection. Both are now living with a sinus tract.

In the remaining 35 patients, one patient underwent debridement, and re-reinfection occurred. Due to reduced general conditions, no further operation could be performed. Rigorous controls (every 3 months) were done. Follow up after reimplantation (2^nd^ revision) was 20 months. In this period, no exacerbation of the infection took place.

In 18 cases (47%), a one stage revision was performed for the second revision surgery. Re-reinfection was present in 44% of cases (CI 95%: 22–69). The cumulative incidence analysis showed a re-reinfection probability after a one stage procedure of 17% (CI 95%: 0–32%) at one year, 33% (CI 95%: 8–52%) at two years, 33% (CI 95%: 8–52%) at five years, 42% (CI 95%: 11–62%) at 10 years, and 42% (CI 95%: 11–62%) at 15 years. One patient died from heart failure and three from metastatic disease.

In 11 cases (31%), a two stage revision was performed for the second revision surgery. Re-reinfection occurred in 55% of cases (95% CI: 31–91). After a two stage procedure, the cumulative incidence analysis showed a re-reinfection probability of 30% (CI 95%: 0–53%) at one year, 42% (CI 95%: 0–66%) at two years, and 78% (CI 95%: 0–96%) at five years. The longest follow-up was 5.04 years; therefore, no estimates for longer follow up were possible. Cumulative incidence curves of re-reinfection did not differ significantly between one and two stage revision (Gray’s test; p = 0.31).

Five patients (13.2%) were treated with an amputation because of poor soft tissue coverage. No further infection occurred in this cohort. One patient died from metastatic disease after 24 months. There were no statistically significant differences in re-reinfection rates between the different surgical procedures (Chi-squared test, p = 0.30).

The detailed outcome of the thirty-eight reinfections is shown in Table 5. One patient died after 59 infection free months for a reason unrelated to infection after two one stage revisions and one two stage revision. Another patient, who was first treated with one stage and then with two stage revision, died from metastatic disease one month after re- reinfection (32 months after last event) with an infected megaprosthesis still *in situ*.

**Table 5:** Outcome of the 38 patients with reinfection. In this cohort, seven patients died for a reason not related to infection.

	Treatment (n) (2nd infection)	Outcome	Treatment (n) (3rd infection)	Outcome	Treatment (n) (4th infection)	Outcome
Reinfection: 38	conservative: 3	infection suppression				
	debridement: 1	3rd infection	conservative: 1	infection-suppression		
	1-stage: 18	success: 10 (4 died)				
		3rd infection: 8	conservative: 1	infection suppression		
			1-stage: 2	success: 1		
				4th infection: 1	1-stage	success
			2-stage: 5	success: 3 (1 died)		
				4th infection: 2	amputation	success
	2-stage: 11	success: 5 (1 died)				
		3rd infection: 6	conservative: 1	infection suppression		
			fistula excision + irrigation-suction drainage: 1	success		
			amputation: 3	success		
			death: 1			
	amputation: 5	success: 5 (1 died)				

### Two-stage revision

**Fig 4:**
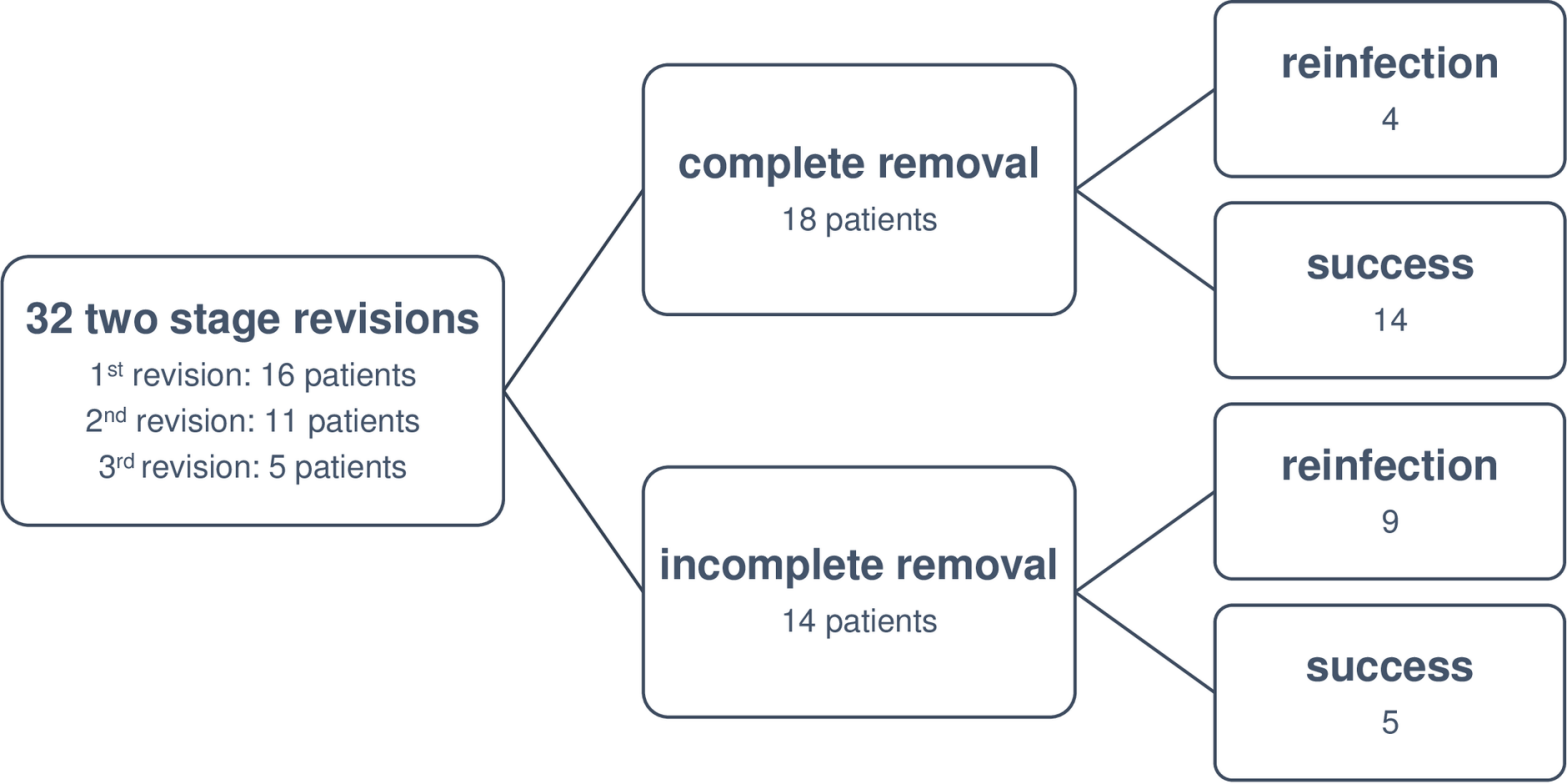
Flow diagram of all patients treated with a two stage revision during the entire study period.

In the entire study period, two stage revision was performed 32 times (Fig 4). In 14 patients (44%) at least one well fixed stem was retained in situ; however, 18 patients (56%) underwent complete removal of the megaprosthesis. The overall reinfection rate was 41% (n = 13/32). In patients with complete removal, the reinfection rate was only 22% (n = 4/18); in patients with a retained stem, the rate was 64% (n = 9/14). The difference in infection rates between patients treated with complete removal of the megaprosthesis and patients with at least one well fixed stem was statistically significant (Fisher’s exact test, p = 0.029).

Analysing one stage revisions and two stage revisions with incomplete removal of the megaprosthesis together as one group (n = 96 [one stage: 82; two stage with incomplete removal: 14]; reinfections: 48 [one stage: 39; two stage with incomplete removal: 9]) over the whole study period, there was a statistical significant difference (Fisher’s exact test, p = 0.039) between this group and patients treated with a two stage revision and complete removal of the stems (n = 18; reinfections: 4).

### Silver-coated megaprosthesis

Overall, an uncoated megaprosthesis was used in 101 revisions (1^st^ revision [n = 73], 2^nd^ revision [n = 23], 3^rd^ revision [n = 5]). Of these, a reinfection occurred 53 (52%) times. A silver-coated prosthesis was used in ten revisions (1^st^ revision [n = 4], 2^nd^ revision [n = 5], 3^rd^ revision [n = 1]) and showed a reinfection in five (50%) cases. There was no statistically significant difference between the titanium group and the silver-coated group (Fisher’s exact test, *p = 1*.*000*) in our study cohort.

### Amputation

Overall, 13 (2%) patients underwent an amputation due to infection after a mean of 2.2 (range: 1–4) infections, and previously 1.2 revisions (range: 0–3). In three patients, amputation due to infection was indicated, but the patients declined amputation. All had a chronic infection with a fistula. If these patients were categorized as amputation, the amputation rate was 3%.

## Discussion

The introduction of chemo- and radiotherapy, new surgical techniques, and modular prosthetic design has led limb salvage to become standard in the treatment of musculoskeletal malignancies [2, 12, 20, 22–25]. Nevertheless, failures such as aseptic loosening, mechanical failure or infection can occur [26–28]. However, the most devastating complication regarding megaprosthesis failure is infection. In our study cohort, the infection rate was calculated at 13%, which is in line with the 8 to 15% reported in the literature [4–9]. If an infection occurs, the probability of reinfection increases dramatically [11]. Therefore, a good surgical strategy for infection eradication is necessary. One stage revision and two stage revision were shown to be promising surgical treatment options for limb salvage in oncological patients to control deep infections [12, 19–21, 25]. Therefore, we aimed to evaluate the performance of one stage and two stage revision in patients after septic modular megaprosthesis and compared both methods to each other.

This study is certainly limited by the retrospective design. Furthermore, the number of oncological patients treated with megaprostheses who experienced an infection is low, especially for patients treated with two stage revision. Nevertheless, this retrospective series represents the largest single-centre observation of primary modular megaprostheses with similar design in the lower limb. Jeys et al. [12] on the other hand also observed patients treated with diaphyseal replacement and hemiprosthesis. It is well known that these kinds of replacements have a lower reinfection rate.

In this series, the reinfection and re-reinfection rate of the limb salvage procedures was 46% and 43%, respectively, which is similar to that reported (43%) by Capanna et al [11]. Table 6 shows a comparison of the literature regarding reinfection rate after one stage revisions. Our incidence of reinfection (49%) was similar to the series by Jeys et al. [12], but slightly higher than in the studies conducted at our institution by Holzer et al. [25] and Funovics et al [20]. All patients of the two latter groups were included in our study. The slightly higher reinfection rate could be due to the longer mean follow-up in our series.

**Table 6:** Comparison of the literature of the reinfection rates (RR) after 1-stage procedures. The follow-up period started after the first revision (m = months).

References	PJI (n)	1-stage	reinfection	RR	Follow up
Holzer et al [25]	19	18	4	22%	52m
Jeys et al [12]	136	33	19	58%	24m
Funovics et al [20]	12	8	3	37%	54m
Present study	83	61	30	49%	87m
Total	250	120	56	47%	54m

Compared to the other limb salvage procedures (Table 2), two stage revision showed the lowest reinfection rate (38%) which is slightly higher than the results reported in the literature (Table 7).

**Table 7:** Comparison of the literature of the reinfection rates (RR) after 2-stage procedures. The follow-up period started after the first revision (m = months). In the series of Flint et al. and Grimer et al. (*patient survival 109m), it is not clear at which point in time follow up began.

References	PJI (n)	2-stage	reinfection	RR	Follow up
Jeys et al [12]	136	58	16	28%	24m
Flint et al [19]	15	11	3	27%	52m
Grimer et al [21]	-	34	10	26%	*
Bindiganavile et al [29]	-	36	8	22%	34m
Present study	83	16	6	38%	46m
total	-	155	43	28%	38m

A possible explanation could be that the underlying study also includes cases in which not all anchorage stems were removed. However, considering only patients treated with complete removal of the megaprosthesis, a lower reinfection rate of only 22% was calculated. These findings are in line with the demonstrated reinfection rates in the literature [12, 19, 21, 29]. On the other hand, the reinfection rate after a two stage revision with retention of a well fixed stem was 64%, which is in line with the reinfection rate after one stage revision (58%) demonstrated by Jeys et al [12]. Therefore, we compared both groups (with and without complete removal) among each other and found a statistically significant difference between both groups (p = 0.029). Based on this information, a comparison between patients treated with one stage revision or two stage revision with at least one anchorage stem and patients treated with complete removal of the megaprosthesis were done. Here, a statistically significant difference was also demonstrated (p = 0.039). Therefore, positive results can only be expected if all components are removed. Otherwise, a two stage revision with incomplete removal of the stems is not superior to a one stage revision (same extension of debridement). In addition, a second operation has the potential risk of a new infection caused by a phenotypically different microorganism. Hence, in cases in which a second operation would be detrimental (e.g. high-risk patients with reduced general conditions), a one stage revision might be the better choice though bearing a higher risk of reinfection.

Due to our investigation, two stage revision with complete removal of the megaprosthesis offers the best infection cure rates and should be first choice for the treatment of infected megaprostheses. One stage revision may only be considered when the microorganism is known and not difficult–to—treat (difficult–to–treat pathogens: resistant to biofilm-active antimicrobials: rifampin-resistant staphylococci, ciprofloxacin-resistant gram-negative bacteria or fungi) with good soft tissue coverage. Nevertheless, one stage revision must include a thorough debridement and an exchange of all components comprising well fixed stems. Exceptionally, a one stage revision with retention of well osseointegrated stems (retention of the prosthesis and exchange of mobile parts) may be performed when the biofilm is still immature (acute PJI: < 4 weeks) or as a palliative intervention with the possibility of applying a chronic fistula in patients with high comorbidities.

Some studies have shown that the use of silver-coated megaprosthesis leads to a reduced rate of infections after primary implantation (in the absence of prior infection) [30–32]. However, the benefits of silver coating in infected revision surgery are still unproved [33]. In a review by Schmidt-Braekling [34], the authors suggest that the protein-related inactivation of silver in postoperative hematoma or wound heling disorders may lead to increased bacterial colonization of the soft tissue, and at these points the silver will not provide sufficient protection against infections. Therefore, it seems that silver-coating megaprostheses show no benefits in infected revision surgery, which is in line with our results. Nevertheless, future studies will be needed to elucidate the effectiveness of silver.

## Conclusion

Infections following resection of bone tumours and reconstruction by megaprostheses are difficult to treat and show high reinfection and re-reinfection rates with substantial risk of amputation. Two stage revision with complete removal of the megaprosthesis (including anchorage stems) showed the best result among limb salvage procedures.
